# Volume of oxygen administered during mechanical ventilation predicts mortality in ICU patients

**DOI:** 10.1186/s13054-023-04499-2

**Published:** 2023-06-19

**Authors:** C. C. A. Grim, L. I. van der Wal, J. A. Bouwens, D. J. van Westerloo, E. de Jonge, H. J. F. Helmerhorst

**Affiliations:** 1 Department of Intensive Care, Leiden University Medical Center, Leiden, The Netherlands; 2 Department of Anesthesiology, Leiden University Medical Center, Leiden, The Netherlands; 3 PrioCura Psychiatry, Rotterdam, The Netherlands

The appropriate administration of oxygen to mechanically ventilated patients in the ICU remains a challenge. While clinical guidelines advocate for conservative oxygenation targets, recent trials have produced conflicting results [1, 2]. The use of different surrogates to assess oxygen exposure and oxygenation, along with confounding by indication, may explain the heterogeneity in findings. We aim to explore a novel parameter of cumulative oxygen exposure, the volume of oxygen administered during mechanical ventilation (MV). We hypothesize that this parameter is a more precise and direct measure of oxygen exposure than previously used surrogates and therefore maybe more reliably linked to outcome. We performed a cohort study using patient data from a tertiary ICU in the Netherlands and included hourly MV settings, arterial blood gas analyses, outcome and demographic data for all patients admitted to the ICU from July 2011 to September 2015.

The volume of oxygen administered to each patient during MV was calculated by estimating the area under the curve of the product of FiO_2_ and ventilatory minute volume as a function of MV time in minutes (FiO_2_ * ventilatory minute volume (L/min) * MV time (minutes)). The result was a metric of total oxygen volume in liters administered to the patient during invasive MV (cumulative oxygen volume). Because this metric was strongly confounded by the duration of ventilation (high level of collinearity, Pearson’s *r* = 0.93), we calculated a time-weighted metric by dividing cumulative oxygen volume by duration of MV (oxygen volume per minute). Patients were categorized into three MV time categories: Patients ventilated for less than 24 h, 24–96 h, and 96 h or longer. The primary outcome of interest was hospital mortality and a logistic regression model was used to analyze the association, adjusted for age, sex, APACHE III score and ventilator time categories. To account for a possible difference of effect size of oxygen volume per minute across MV time categories, we included an interaction term in the adjusted model (ventilatory time categories * oxygen volume per minute). The validity of the prediction model was evaluated by comparing it with logistic regression models of SpO_2_, PaO_2_ and PaO_2_ /FiO_2_ ratio for hospital mortality and a Nagelkerke *R*^2^ was determined.

5017 eligible patients were included. Compared to non-surviving patients, surviving patients were younger and had lower APACHE III scores, higher SpO_2_, higher PaO_2_, higher PaO_2_/FiO_2_ ratio, lower oxygen volume, shorter MV time and shorter ICU length of stay. Oxygen volume per minute was significantly associated with hospital mortality after adjustment for APACHE III score and MV time (OR 2.2 (95% C.I. 1.9–2.4) (Table 1). The interaction term of ventilatory time categories and oxygen volume per minute was not significantly associated with hospital mortality (OR 1.00 (95% C.I. 0.75–1.34), and OR 1.01 (95% C.I. 0.76–1.34), for MV time 24 h compared to ventilation 24–96 h and > 96 h, respectively). Both SpO_2_ and PaO_2_/FiO_2_ ratio models were associated with hospital mortality. Nagelkerke *R*^2^ for the PaO_2_/FiO_2_ ratio with hospital mortality model was 0.53, for the SpO_2_ model 0.53 as well, and for the oxygen volume model 0.58. A detailed description of the methods and results is provided in Additional file 1.

**Table 1:** Logistic regression model of hospital mortality and oxygen volume per minute

	OR (95% C.I.)	*P* value
*Crude model*		
Oxygen volume per minute	3.26 (2.96–3.60)	< 0.001
*Adjusted model*		
Oxygen volume per minute	3.62 (3.27–4.03)	< 0.001
*Fully adjusted model*		
Oxygen volume per minute	2.15 (1.91–2.43)	< 0.001
*Fully adjusted model with interactions terms*		
Oxygen volume per minute	2.15 (1.83–2.54)	< 0.001
Ventilatory time 24–96 h	2.21 (1.07–4.48)	0.03
Ventilatory time > 96 h	2.82 (1.32–5.91)	0.007
Oxygen volume per minute * Ventilatory time 24–96 h	1.00 (0.75–1.34)	0.98
Oxygen volume per minute * Ventilatory time > 96 h	1.0 (0.8–1.3)	0.97

*SE* standard error, *OR* odds ratio, *C.I.* confidence interval. Oxygen volume per minute was calculated by dividing cumulative oxygen volume by MV time. Adjusted model: adjusted for age and sex. Fully adjusted model: adjusted for age, sex, ventilatory time categories and APACHE III score. Fully adjusted model with interaction terms: adjusted for age, sex, ventilator time categories, APACHE III score and included interaction term (ventilatory time categories* oxygen volume per minute). APACHE: Acute Physiology and Chronic Health Evaluation

This cohort study analyzed patient data from one ICU in the Netherlands to investigate the association between oxygen exposure during mechanical ventilation (MV) and hospital mortality. Oxygen volume per minute administered during MV was independently associated with hospital mortality, with a change of 1 L per minute in oxygen volume per minute increasing the OR for hospital mortality by a factor of 3.26. The effect of oxygen volume per minute of oxygen on in-hospital mortality was not different across ventilator time categories, proposing an effect of oxygen exposure independent of ventilation time on mortality. If our findings are the result of a causal relationship between oxygen volume and mortality, it suggests direct toxic effects of oxygen and its supplemental use. The volume of administered oxygen was a stronger predictor of hospital mortality compared to existing parameters of oxygen exposure.

The study has several strengths, including the development of a novel and more accurate measure of oxygen exposure, a comprehensive dataset consisting of complete hourly data of the mechanically ventilated period per individual patient admitted to the ICU over a 4-year period, and the automatic extraction of data from the patient data management system. However, the study also has limitations, including its observational nature, residual confounding, the single-center dataset, and the lack of control for specific diagnosis.

In conclusion, oxygen volume per minute is a stronger predictor of mortality than established oxygen metrics. Therefore, oxygen volume per minute administered during MV seems to be a reliable parameter of oxygen exposure. Previously used oxygenation parameters may not completely capture the direct effect on outcome of exposure to oxygen as a vital but potentially toxic agent. Future studies should evaluate the replicability of our study's results.

## Supplementary Information

**Additional file 1:** Detailed description of the methods and results.

## Data Availability

A more detailed description of the methods and results was added as Additional file 1. The datasets supporting the conclusions of this article are not publicly available but are available from the corresponding author on reasonable request.

## Abbreviations

95% C.I.: 95% Confidence intervals
APACHE: Acute Physiology and Chronic Health Evaluation
FiO_2_: Fraction of inspired oxygen
ICU: Intensive care unit
L: Liters
MV: Mechanical ventilation
OR: Odds ratio
PaO_2_: Partial pressure of arterial oxygen
SpO_2_: Peripheral oxygen saturation
