# Supplementary material for: Volume of oxygen administered during mechanical ventilation predicts mortality in ICU patients
Source: Crit Care. 2023 Jun 19;27:242. doi: 10.1186/s13054-023-04499-2 (PMC10278334; doi:10.1186/s13054-023-04499-2)
Supplement: Supplementary file 1 — Additional file 1: Detailed description of the methods and results. [file 13054_2023_4499_MOESM1_ESM.docx]

Additional material:

Volume of oxygen administered during mechanical ventilation predicts mortality in ICU patients

C.C.A. Grim^1,2*^, L.I. van der Wal^1^, J.A. Bouwens^3^, D.J. van Westerloo^1^, E. de Jonge^1^, H.J.F. Helmerhorst^1,2^

* Corresponding author: c.c.a.grim@lumc.nl

# ^1^Department of Intensive Care, Leiden University Medical Center, Leiden, The Netherlands

^2^Department of Anesthesiology, Leiden University Medical Center, Leiden, The Netherlands

^3^PrioCura Psychiatry, Rotterdam, The Netherlands

Methods

This cohort study was performed using patient data from one tertiary ICU in the Netherlands. Anonymous encrypted data were extracted from the patient data management system (PDMS, MetaVision, iMDsoft, Leiden, The Netherlands), according to a previously described method (1). All patients admitted to the ICU from July 2011 to September 2015 that were invasively mechanically ventilated were included in the database. The database included hourly observations of MV settings per patient during the time of MV. The dataset was supplemented with arterial blood gas analyses, outcome and demographic data from a previously used dataset (1-3). Outcome and demographic data were previously extracted from the Dutch National Intensive Care Evaluation (NICE) registry (19), a high quality database, which enables to quantify and improve the quality of ICU care by offering feedback and benchmarking on patient outcomes and process indicators. Patients were excluded when more than three consecutive hourly ventilation data points were missing (e.g., patient transport, intervention or diagnostics at non-ICU location). Informed consent or approval by an ethical committee was not needed according to the Dutch Medical Research Involving Human Subjects Act as only non-identifying routinely registered data was used.

To obtain a robust measure for the exposure to oxygen, we constructed a metric for volume of oxygen administered during MV. In each subject, this metric was calculated by estimating the area under the curve (AUC) of the product of FiO_2_ and ventilatory minute volume as a function of MV time in minutes (FiO_2_ * ventilatory minute volume (L/min) * MV time (minutes)). The result was a metric of total oxygen volume in liters (L) administered to the patient for the duration of invasive MV (cumulative oxygen volume). Because this metric may be strongly confounded by the duration of ventilation (high level of collinearity, Pearson’s r = 0.93), we calculated a time weighted metric. The average volume of oxygen received per minute was calculated by dividing cumulative oxygen volume by the duration of MV (oxygen volume per minute). We divided our cohort into three MV time categories: patients ventilated for less than 24 hours, 24-96 hours, and for 96 hours or longer. The interaction was assessed between MV time categories and oxygen volume per minute. We were then able to investigate the effect size of exposure to oxygen by MV and control for the effects of a longer time of ventilation.

Normally distributed data were described as mean with standard deviation (SD). Non-normally distributed data are described as median with interquartile range (IQR). Categorical variables are summarized as percentages. Between group differences were analyzed using student-t-test and Mann-Whitney U test, for normally and non-normally distributed data, respectively. Categorical variables were compared using the chi-square test.

The primary outcome of interest was hospital mortality. In the crude logistic regression model (Model 1), we analyzed the association between oxygen volume per minute and hospital mortality. In Model 2, we additionally adjusted for important confounders age and sex. In the fully adjusted model (Model 3), we included other major variables that were considered to be in the causal pathway, including oxygen volume per minute, ventilatory time categories and APACHE III score. To account for a possible difference of effect size of oxygen volume per minute across MV time categories, we included an interaction term in the fully adjusted model (ventilatory time categories* oxygen volume per minute) (Model 4). Furthermore, a subgroup analysis was performed by admission type: emergency, medical or surgical. Results were expressed as odds ratios (OR) with 95% confidence intervals (95% C.I.). The validity of the oxygen volume per minute prediction model of hospital mortality was evaluated by comparing the model with logistic regression models of SpO_2_, PaO_2_ and PaO_2_/FiO_2_ ratio for hospital mortality. For each oxygenation parameter (SpO_2_, PaO_2_ and PaO_2_/FiO_2_ ratio) an AUC was estimated over the MV time. The average per minute for each oxygenation parameter was calculated over the MV time by dividing the AUC by the MV time. Nagelkerke R^2^ was determined for these models and the oxygen volume per minute model for hospital mortality. To compare the coefficients of the different parameters, the coefficient ‘B’ was converted to a ‘beta’ by using standardized data (i.e. a Z-score) in the model.

Missing data for single data points of oxygenation parameters were imputed with the previous observation under the assumption that data was missing at random. A sensitivity analysis confirmed that patients with missing data points did not differ significantly from patients without missing data regarding age, sex, APACHE III score, and mortality (data not shown). Missing outcome and demographic data from the previously used dataset was not imputed. A P-value of <0.05 was considered statistically significant. Statistical analyses were performed using Rstudio 2022.07.2 with R 4.1.0.

# Results

In total, 5017 eligible patients were mechanically ventilated between July 2011 and September 2015 (Figure 1). Most patients in this cohort were male and most patients were admitted to the ICU for medical reasons. Compared to non-surviving patients, surviving patients were younger, had lower APACHE III scores, higher SpO_2_, higher PaO_2_, higher PaO_2_/FiO_2_ ratio, lower oxygen volume, shorter MV time and shorter ICU length of stay (LOS). Complete patient characteristics are listed in Table 1.

In this cohort, 3773 (75.2%) patients were mechanically ventilated shorter than 24 hours, 688 (13.7%) were mechanically ventilated between 24 and 96 hours, and 556 (11.1%) for longer than 96 hours. Oxygen volumes according to MV time are provided in Table 2.

Table 3 shows the results from the regression analyses. The effect of oxygen volume per minute was attenuated but remained significantly associated with hospital mortality after adjustment for APACHE III score and MV time (Model 3: OR 2.2 (95%C.I.: 1.9-2.4). The interaction term of ventilatory time categories and oxygen volume per minute was not significantly associated with hospital mortality (Model 4: OR 1.00 (95%C.I.: 0.75-1.34), and OR 1.01 (95%C.I.: 0.76-1.34), for MV time 24 hours compared to ventilation 24-96 hours and >96 hours, respectively). In a subgroup analysis by admission type there was no significant attenuation of the association between oxygen volume per minute and hospital mortality.

The regression model of PaO_2_ was not significantly associated with hospital mortality (Table 4). Both SpO_2_ and PaO_2_/FiO_2_ ratio models were associated with hospital mortality. Nagelkerke R^2^ for the PaO_2_/FiO_2_ ratio with hospital mortality model was 0.53, for the SpO_2_ model 0.53 as well, and for the oxygen volume model 0.58 (Table 5).

# References

1. Helmerhorst HJ, Schultz MJ, van der Voort PH, Bosman RJ, Juffermans NP, de Wilde RB, et al. Effectiveness and Clinical Outcomes of a Two-Step Implementation of Conservative Oxygenation Targets in Critically Ill Patients: A Before and After Trial. Crit Care Med. 2016;44(3):554-63.
2. Helmerhorst HJ, Schultz MJ, van der Voort PH, Bosman RJ, Juffermans NP, de Jonge E, et al. Self-reported attitudes versus actual practice of oxygen therapy by ICU physicians and nurses. Ann Intensive Care. 2014;4:23.
3. Grim CC, Helmerhorst HJ, Schultz MJ, Winters T, van der Voort PH, van Westerloo DJ, et al. Changes in Attitudes and Actual Practice of Oxygen Therapy in ICUs after Implementation of a Conservative Oxygenation Guideline. Respiratory Care. 2020:respcare.07527

Figure 1. Flowchart of patient selection

N= 6950

Patients admitted to the ICU from July 2011-September 2015 with hourly observations of MV settings

- N=927

Missing datapoints (one or more data points were more than three hours apart)

- N=1006

Missing outcome data

N= 5017

Patients included for analysis

| **Table 1. Characteristics of included patients** | | | | |  |
| --- | --- | --- | --- | --- | --- |
|  | **All n=5017** | **Survivors n=4246 (84.6%)** | **Non-survivors n=771 (15.4%)** | **P-value** |  |
| **Baseline characteristics** | | | | |  |
| Age, years | 66 (55-73) | 65 (55-73) | 67 (57-75) | <0.001 |  |
| Gender, n (%) female | 1713 (34.1) | 1414 (33.3) | 299 (38.7) | <0.014 |  |
| Admission type, n(%) |  |  |  | <0.001 |  |
| Medical | 3711 (73.9) | 3009 (70.9) | 702 (91.1) |  |  |
| Emergency surgical | 196 (3.9) | 161 (3.8) | 35 (4.5) |  |  |
| Planned surgical | 1110 (22.1) | 1076 (25.3) | 34 (4.4) |  |  |
| APACHE III score | 54 (40-76) | 50 (38-65) | 110 (85-135) | <0.001 |  |
| **Vital signs and laboratory data over entire admission** | | | | |  |
| SpO_2_, % | 98 (97-100) | 99 (97-100) | 96 (95-98) | <0.001 |  |
| PaO_2_, mmHg | 98 (82-124) | 101 (84-128) | 88 (77-108) | <0.001 |  |
| **MV settings over entire admission** | | | | |  |
| FiO_2_, % | 40 (35-40) | 40 (33-40) | 40 (35-58) | <0.001 |  |
| PEEP, cmH_2_O | 5 (5-6) | 5 (5-5) | 7 (5-9) | <0.001 |  |
| Ventilatory minute volume, liters/min | 8.9 (2.5) | 8.9 (2.2) | 11.1 (3.3) | <0.001 |  |
| PaO_2_ to FiO_2_ ratio | 278 (101) | 290 (97) | 224 (101) | <0.001 |  |
| Cumulative oxygen volume, liters | 677 (323-2802) | 554 (288-1421) | 7216 (2165-19762) | <0.001 |  |
| Oxygen volume per minute, liters | 1.8 (1.4-2.3) | 1.7 (1.3-2.1) | 2.7 (1.9-3.7) | <0.001 |  |
| **Outcomes** | | | | |  |
| MV time, hours | 6.5 (3.6-18.6) | 5.8 (3.4-12.4) | 26.5 (7.7-75.9) | <0.001 |  |
| ICU length of stay, hours | 24 (20-66) | 23 (20-49) | 56 (23-152) | <0.001 |  |
| APACHE: Acute Physiology and Chronic Health Evaluation. Data are means (standard deviation (SD)) or median (interquartile range (IQR), unless stated otherwise. Cumulative oxygen volume was calculated as the area under the curve with the product of FiO2 and ventilatory minute volume on the y-axis and MV time on the x-axis. Oxygen volume per minute was calculated by dividing total oxygen volume by MV time. | | | | |  |
|  |  |  |  |  |  |
|  |  |  |  |  |  |

| **Table 2. Oxygen volume by ventilation and admission time** | | | | |  |
| --- | --- | --- | --- | --- | --- |
|  | **Ventilation time** | | |  |  |
|  | **< 24 hours n= 3773 (75.2%)** | **24-96 hours n= 688 (13.7%)** | **>96 hours n= 556 (11.%)** | **P-value** |  |
| **Cumulative oxygen volume, liters** | 474 (252-832) | 5648 (3591-8767) | 24829 (16041-8767) | <0.001 |  |
| **Oxygen volume per minute, liters** | 1.7 (1.3-2.1) | 2.0 (1.5-2.6) | 2.3 (1.8-3.0) | <0.001 |  |
|  |  | **ICU admission time** |  |  |  |
|  | **<24 hours n=2454 (49.1%)** | **24-96 hours n=1702 (34.1%)** | **>96 hours n=842 (16.9%)** |  |  |
| **Cumulative oxygen volume, liters** | 411 (232-25710) | 1036 (437-2902) | 15313 (7338-32024) | <0.001 |  |
| **Oxygen volume per minute, liters** | 1.7 (1.3-2.1) | 1.8 (1.4-2.3) | 2.1 (1.6-2.7) | <0.001 |  |
| Data are presented as median (interquartile range (IQR)). The differences between the three groups were compared by the one way ANOVA test. Cumulative oxygen volume was calculated as the area under the curve with the product of FiO2 and ventilatory minute volume on the y-axis and MV time on the x-axis. Oxygen volume per minute was calculated by dividing total oxygen volume by MV time. | | | | |  |
|  |  |  |  |  |  |
|  |  |  |  |  |  |
|  |  |  |  |  |  |
|  |  |  |  |  |  |

| **Table 3. Logistic regression model of hospital mortality and oxygen volume per minute** | | |
| --- | --- | --- |
|  | **OR (95% C.I.)** | **P-value** |
| **Model 1 (crude association)** | | |
| Oxygen volume per minute | 3.26 (2.96-3.60) | <0.001 |
| **Model 2 (adjusted model)** | | |
| Oxygen volume per minute | 3.62 (3.27-4.03) | <0.001 |
| **Model 3 (fully adjusted model)** | | |
| Oxygen volume per minute | 2.15 (1.91-2.43) | <0.001 |
| **Model 4 (fully adjusted model with interactions terms)** | | |
| Oxygen volume per minute | 2.15 (1.83-2.54) | <0.001 |
| Ventilatory time 24-96 hours | 2.21 (1.07-4.48) | 0.03 |
| Ventilatory time >96 hours | 2.82 (1.32-5.91) | 0.007 |
| Oxygen volume per minute * Ventilatory time 24-96 hours | 1.00 (0.75-1.34) | 0.98 |
| Oxygen volume per minute * Ventilatory time >96 hours | 1.0 (0.8-1.3) | 0.97 |
| SE: standard error. OR: odds ratio. C.I.: confidence interval. APACHE: Acute Physiology and Chronic Health Evaluation.  Oxygen volume per minute was calculated by dividing cumulative oxygen volume by MV time. | | |
|  |  |  |
|  |  |  |
|  |  |  |

| **Table 4. Logistic regression model for hospital mortality** | | | | | | | | | |  |
| --- | --- | --- | --- | --- | --- | --- | --- | --- | --- | --- |
|  | **PaO_2_** | | | **PaO_2_/FiO_2_** | | | **SpO_2_** | | |  |
| **Fully adjusted model** | **B (SE)** | **OR (95% C.I)** | **P-value** | **B (SE)** | **OR (95% C.I)** | **p-value** | **B (SE)** | **OR (95% C.I)** | **p-value** |  |
| Constant | -5.79 (0.38) | 0.00 (0.00-0.01) | <0.001 | -4.28 (0.39) | 0.01 (0.01-0.03) | <0.001 | 7.49 (1.86) | 1782.25 (48.10-71589.47) | <0.001 |  |
| Oxygen related parameter (PaO_2_, PaO_2_/FiO_2_, SpO_2_) | 0.00 (0.00) | 1.00 (1.00-1.01) | 0.16 | 0.00 (0.00) | 0.99 (0.99-0.99) | <0.001 | -0.13 (0.02) | 0.87 (0.84-0.91) | <0.001 |  |
| Age | 0.00 (0.00) | 1.00 (0.99-1.00) | 0.28 | -0.010(0.00) | 0.99 (0.98-0.99) | 0.03 | -0.01 (0.0) | 0.99 (0.99-1.00) | 0.19 |  |
| Female sex | 0.25 (0.11) | 1.28 (1.02-1.60) | 0.03 | 0.43 (0.12) | 1.53 (1.21-1.94) | <0.001 | 0.28 (0.12) | 1.32 (1.05-1.66) | 0.02 |  |
| APACHE score | 0.05 (0.00) | 1.05 (1.04-1.05) | <0.001 | 0.05 (0.00) | 1.05 (1.04-1.05) | <0.001 | 0.05 (0.00) | 1.05 (1.04-1.05) | <0.001 |  |
| Ventilatory time >96 hours | 1.22 (0.15) | 3.39 (2.52-4.55) | <0.001 | 1.02 (0.15) | 2.77 (2.06-3.72) | <0.001 | 1.16 (0.15) | 3.18 (2.39-4.22) | <0.001 |  |
| Ventilatory time 24-96 hours | 0.72 (0.14) | 2.05 (1.54-2.71) | <0.001 | 0.56 (0.15) | 1.75 (1.31-2.32) | <0.001 | 0.68 (0.15) | 1.97 (1.48-2.62) | <0.001 |  |
| SE: standard error. OR: odds ratio. C.I.: confidence interval. APACHE: Acute Physiology and Chronic Health Evaluation. PaO2, PaO2/FiO2 and SpO2 were calculated as weighted average over the mechanically ventilated time | | | | | | | | | |  |
|  |  |  |  |  |  |  |  |  |  |  |

| **Table 5. Logistic regression model and Nagelkerke R2 for oxygen volume per minute, SpO_2_, PaO_2_ to FiO_2_ ratio and PaO_2_** | | | | | | | |  |  |
| --- | --- | --- | --- | --- | --- | --- | --- | --- | --- |
|  | **Oxygen volume per minute** | **P-value** | **PaO_2_** | **P-value** | **PaO_2_/FiO_2_** | **P-value** | **SpO_2_** | **P-value** |  |
| **OR (95%C.I.)** | 2.05 (1.84-2.31) | <0.001 | 1.09 (0.97-1.23) | 0.16 | 0.70 (0.61-0.80) | <0.001 | 0.69 (0.62-0.76) | <0.001 |  |
| **Nagelkerke R2** | 0.58 |  | 0.52 |  | 0.53 |  | 0.53 |  |  |
| SE: standard error. OR: odds ratio. C.I.: confidence interval. PaO_2_, PaO_2_/FiO_2_ and SpO_2_ were calculated as weighted average over the mechanically ventilated time. Oxygen volume per minute was calculated by dividing cumulative oxygen volume by MV time. PaO_2_, PaO_2_/FiO_2_ and SpO_2_ were calculated as weighted average over the mechanically ventilated time | | | | | | | | |  |
|  |  |  |  |  |  |  |  |  |  |
|  |  |  |  |  |  |  |  |  |  |
